# Multi-shape active composites by 3D printing of digital shape memory polymers

**DOI:** 10.1038/srep24224

**Published:** 2016-04-13

**Authors:** Jiangtao Wu, Chao Yuan, Zhen Ding, Michael Isakov, Yiqi Mao, Tiejun Wang, Martin L. Dunn, H. Jerry Qi

**Affiliations:** 1 The George Woodruff School of Mechanical Engineering, Georgia Institute of Technology, Atlanta, GA 30332, USA; 2 State Key Laboratory for Strength and Virbration of Mechanical Structures, Department of Engineering Mechanics, Xi’an Jiaotong University, Xi’an 710049, China; 3 SUTD Digital Manufacturing and Design (DManD) Centre, Singapore University of Technology and Design, Singapore

## Abstract

Recent research using 3D printing to create active structures has added an exciting new dimension to 3D printing technology. After being printed, these active, often composite, materials can change their shape over time; this has been termed as 4D printing. In this paper, we demonstrate the design and manufacture of active composites that can take multiple shapes, depending on the environmental temperature. This is achieved by 3D printing layered composite structures with multiple families of shape memory polymer (SMP) fibers – digital SMPs - with different glass transition temperatures (*T*_*g*_) to control the transformation of the structure. After a simple single-step thermomechanical programming process, the fiber families can be sequentially activated to bend when the temperature is increased. By tuning the volume fraction of the fibers, bending deformation can be controlled. We develop a theoretical model to predict the deformation behavior for better understanding the phenomena and aiding the design. We also design and print several flat 2D structures that can be programmed to fold and open themselves when subjected to heat. With the advantages of an easy fabrication process and the controllable multi-shape memory effect, the printed SMP composites have a great potential in 4D printing applications.

Contemporary 3D printing technologies enable fabrication of components with fine details by accurately placing multiple materials at any position within a design domain. Due to these great advantages, 3D printing is attracting more and more research and development interest, to transform from a prototyping tool to a fabrication powerhouse for applications ranging from tissue engineering to aerospace parts and structures^1^,^2^,^3^,^4^. For example, 3D printing has been used to fabricate microstructures from ceramics^5^ to metals, which provides a new method for manufacturing microelectromechanical systems (MEMS)^6^. 3D printed calcium phosphate and collagen scaffold composites were used for bone regeneration^7^. In addition, NASA has used 3D printing to fabricate some rocket parts, and their tests show that 3D printing can save time and reduce costs by 60% or more^8^. More recently, the emergence of 4D printing, where active materials are used in 3D printing, presents a potentially powerful extension of 3D printing for active structure and device applications^9^,^10^. Ge *et al.* reported 4D printing of active composites where shape memory polymer (SMP) fibers were used to activate the shape change of the printed composites^10^,^11^. Tibbits and his colleagues proposed a new design of complex self-evolving structures by 4D printing hydrophilic materials which can form stretching, folding and bending deformation by swelling in water to create and exploit mismatch strains within a structure^12^. Bakarich *et al.* printed thermally actuating hydrogels that can be used as a valve to control the flow of water by automatically closing upon exposure to hot water and opening in cold water^13^. Zhang *et al.* have reported that they fabricated by 3D printing a heat-shrinkable polymer whose initial microstructure can undergo a spontaneous pattern transformation under heating^14^. Combining these smart materials and the advanced 3D printing technology yields what is referred to as 4D printing and provides tremendous opportunities for designing and fabricating smart or active structures efficiently and easily.

Active materials and structures that can change their properties and shape have great technological potential, e.g., in sensors, robotics, self-packaging devices, aerospace exploring and bio-medical applications^15^,^16^,^17^,^18^. Among active materials, shape memory polymers (SMPs) have been intensively studied because of their ability to generate a large shape change under an external stimuli us, such as temperature^19^,^20^,^21^, light^22^, electric^23^,^24^ and magnetic fields^25^,^26^. Generally, in order to achieve shape memory effects (SMEs), the polymer should have one transition point, such as glass transition temperature, crystal-melt transition, etc. For example, typical amorphous polymers can utilize the glass transition to obtain shape memory effects between two shapes. It is also desirable to develop polymers or composites that can predictably change among multiple different shapes. In order to achieve triple or multi-shape changes in SMPs, two or more reversible transition points are needed. For example, Xie *et al.* reported that they can create triple-shape memory structures by fabricating the composites materials which have well-separated glass transitions points^27^. Luo and Mather developed a shape memory composite with triple-shape memory effects by using glass transition and crystal-melt transition^28^,^29^. Digital materials, a new class of composite materials that are created by 3D printing multiple polymers with different properties in precise geometric configurations that are mixed and cured at different ratios, offer an easy and simple way to fabricate materials with desirable properties by design. Recently, we demonstrated the design of components using digital SMPs that exploit the spatial variation of dynamic mechanical properties to produce shapes in a precisely controlled temporal shape changing sequence when activated by a uniform thermal stimulus^30^,^31^.

In this paper, we demonstrate an approach to design and manufacture flat layered composites consisting of families of digital SMP fibers in a rubbery matrix. After being programmed into a temporary shape by a simple thermal-mechanical training program, the printed composite is able to change into multiple shapes and then recover the flat permanent shape when stimulated by temperature. To understand the shape memory behavior of these composites with multiple active fiber families, we develop a new theoretical model to describe the evolution of curvature during the shape recovery process. The influence of the volume fraction of SMP fibers and the strain on the curvature evolution of the composites is determined by experiments and theoretical modeling. Using the theoretical predictions as a guide, we design and manufacture several self-folding and opening structures that demonstrate the performance afforded by the tremendous design freedom.

## Results

### Design concepts

**Figure 1:**
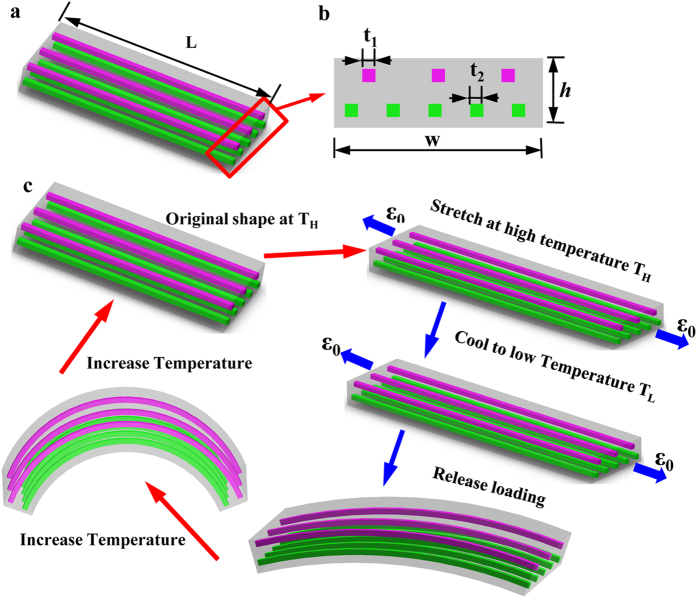
Schematics of the printed SMP composite design. (**a**) The design of the two layer SMP composite strips and (**b**) the characterization of the design. The purple color represents the fiber with higher Tg and the green color represents the fiber with lower Tg. (**c**) The typical programming steps for SMPs and desired response.

The composites in this study consist of three materials with different glass transition temperatures (T_g_s) and are part of the material library of the multi-material 3D printer (Objet260 Connex, Stratasys Inc, Edina, MN, USA). Figure 1a shows the design of a two-layer composite. The matrix is TangoBlack+, which has the lowest T_g_ (~2 °C) of the three materials. Two families of digital SMP fibers with different T_g_s are embedded in the two layers, respectively, with prescribed volume fractions. The fibers (fiber 1: DM8530, T_g_~57 °C; fiber 2: DM9895, T_g_~38 °C,) have shape memory effects in the temperature range between ~20 °C and ~70 °C. The dimensions of the design are annotated in Fig. 1b.

The thermomechanical programming steps are shown in Fig. 1c. We first stretch the composite strip at an elevated programming temperature T_H_, which is higher than T_g_s of both fibers, to a prescribed strain (*ε*_0_), and then cool it to the low temperature 0 °C (T_L_, which is lower than T_g_s of both fiber materials) while maintaining the strain ε_0_. The applied strain is then released, which fixes the composite in the first temporary shape. Upon heating, the strip gains the ability to deform due to the temperature dependent viscoelastic properties of the matrix and fibers; the amount of deformation depends on the thermomechanical properties of the fibers and the matrix (such us T_g_, stiffness and stress relaxation time), the programming strain and the ambient temperature. If we heat the sample to a temperature that is higher than the T_g_ of the matrix but lower than those of the fibers, the sample is able to change to a second temporary shape. Due to the long stress relaxation time of the fibers at low temperatures, we are able to obtain a series of temporary shapes with different bending curvatures if we increase the temperature in a staggered manner. If we heat the sample to the temperature higher than the T_g_s of both fibers, it will recover the flat permanent shape.

### The shape memory behavior of the SMP composite strip

**Figure 2:**
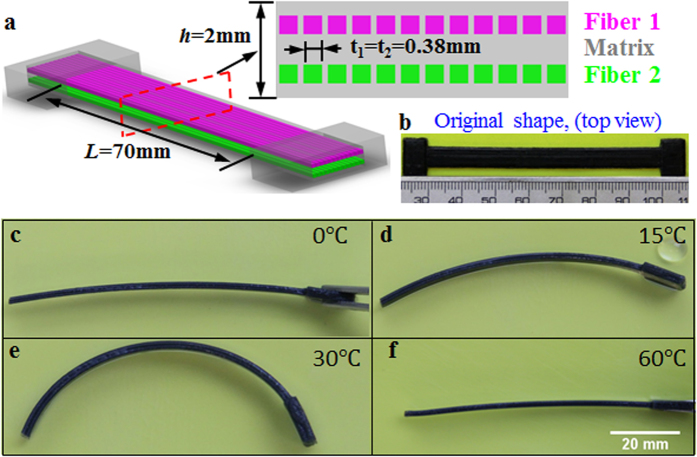
Multi-shape memory effects of a printed active composite strip. (**a**) The design and dimensions of the sample. The enlarged drawing is the cross section of the structure. (**b**) The original printed sample. The length scale in the bottom is in mm. (**c**–**f**) Shape change of the sample at different temperature.

To demonstrate the multi-shape memory effects of the 3D printed SMP composites, we print the active composite strip by following the design concept illustrated above. Here, the strip is 70 mm long, 6 mm wide, and 2 mm thick, with the fiber dimensions of t_1_ =t_2_ = 0.38 mm. The fibers are placed at the center of each layer, i.e., the center of fiber 1 is 0.5 mm from the top surface and the center of fiber 2 is 0.5 mm from the bottom surface. The fibers are uniformly spaced at 0.5 mm (fiber center to fiber center) to give volume fractions of 14% for either fiber 1 or fiber 2. In order to facilitate the stretching of the sample, two handles are directly printed at the ends of the sample, and one handle is cut off before reheating. The printed sample is first stretched at high temperature *T*_*H*_ = 70°C with a strain of 10%, cooled in cold water of ~0 °C, while the loading strain is kept. After the sample is fully cooled down, the strain loading is released. Then the sample is stepwise heated in water with three different temperatures (15 °C, 30 °C, 60 °C) to demonstrate the shape recovery process. Figure 2b shows the top view of the original printed sample. Figure 2c–f show the shape memory effects of the sample. Figure 2c is the temporary shape of the sample at 0 °C after programming. After programming the sample bends slightly towards the side that contains fibers with lower T_g_. When we cool for one minute at the temperature of 15 °C, which is lower than the T_g_ of fiber 2, the sample bends more to form a new temporary shape (Fig. 2d). After that, we heat the sample in 30 °C water for 1 min. Figure 2e shows the new temporary shape of the sample. Comparing the bending angles of the sample in Fig. 2d,e, we can see the bending angle of the sample increases with temperature when the temperature is lower than the T_g_ of fiber 2. Finally we heat the strip to 60 °C and the sample recovers its original flat shape (Fig. 2f). The sequence of transformations of the 3D printed SMP composites indicates that we can achieve multiple shapes using 3D printing technology with a programming process that consists of a single simple step training. In addition, Fig. 2 shows a distinct feature of the new design: unlike conventional SME in polymers where the shape change is simply from one to another; here, a simple stretch can create multiple shape changes, i.e. it folds (bends) first, then unfolds (unbends) to become straight.

**Figure 3:**
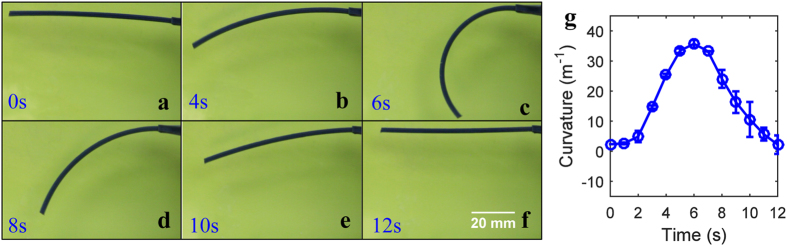
Bending actuation of the printed active composite strip during the recovery process. (**a–f**) Snapshots of the sample bending at different times. All six images are the side view of the sample. (**g**) Variation of the bending curvature during the recovery process.

To further investigate the shape recovery process of the printed active composite (PAC) strip, we directly heat the sample under hot water to the prescribed temperature of T_H_ = 70 °C. A video camera is used to record the entire deformation process. The bending behavior of the sample can be described by the variation of the bending curvature. Figure 3a–f show the deformation behavior of the sample at different times. The curvature of the sample as a function of the time in hot water is plotted in Fig. 3g. It is clear that the curvature first increases when the strip begins to form the new temporary shape and then decreases when the sample begins to recover to its permanent shape after the temperature rises higher than the T_g_s of both fibers. Note that the whole recovery time is only 12 seconds, indicating the fast response speed of the shape change.

The experimental results in Figs 2 and 3 confirm our design concept. A simple explanation can be used to better understand the deformation mechanisms. In the stretching step, since the temperature is higher than the T_g_s of fibers and matrix, all the materials are in the rubbery state, and the high mobility of the polymer chains in the materials allows the chains to rearrange easily. As we cool the sample to a temperature that is below the T_g_s of all the materials, the mobility of the polymer chains significantly decreases and all the materials transform from the rubbery state to the glassy state. As illustrated in previous studies on SMPs^32^, a small recovery is always expected in SMPs; the amount of recovery depends on the thermomechanical properties of the SMP. Therefore, after we release the strain at the temperature below the T_g_s of all the materials, the constituent materials in the strip will recover slightly, but by different amounts, which cause a mismatch strain, and thus bending of the strip. In the recovery step, when we increase the temperature to above the T_g_ of the matrix, the increase chain mobility in the matrix polymer causes the recovery of the matrix, which leads to further bending of the strip. As we further increase the temperature, the mobility of the polymer chains in the fiber 1 polymer (which has a lower T_g_ than fiber 2 polymer) increases, therefore the side of the sample with these fibers can recover more strain, which increases the bending deformation. Finally, after the temperature rises higher than the T_g_s of both fibers, the sample will return to its permanent relaxed shape. In addition, the volume fraction of each family of fibers plays an important role in determining the shape changing behavior of the composite and is an easily controlled design parameter. In the following section, a theoretical model is developed to characterize the recovery process of the sample.

### Theoretical prediction of multi-shape memory behavior

**Figure 4:**
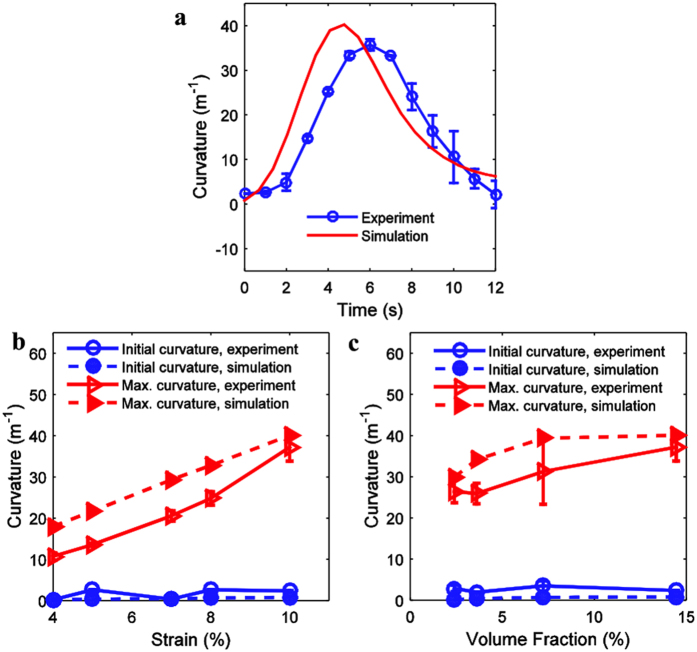
Comparison of the curvatures between experiments and theoretical model. (**a**) Bending curvature of the SMP composite vs heating time. (**b**) Effects of the programming strain and (**c**) the volume fraction of fibers on the initial and maximum curvature.

In order to better understand the deformation behavior of the SMP composites, a theoretical model is developed that can be used to design SMP composites. In this model, the bending behavior of the composite strip is described using classical laminate theory, and a multi-branch model is used to describe the viscoelastic mechanical behavior of the matrix and fibers. Since the recovery speed is relatively fast, the heat transfer problem is considered to capture the non-uniform temperature distribution in the sample during the heating process. The details of the theoretical model are described in the methods section, and the constitutive model for viscoelastic mechanical behavior and the parameter characterization of the printed SMP material are provided in the supplementary information. Using the theoretical model, we can predict the deformation behavior of the SMP composites. The curvature variation of the SMP composites strip is calculated as a function of the heating time. Figure 4a shows the comparison of the experimental and theoretical results of the curvature variation of the PAC sample during the heating process. Overall, the model captures the variation of the sample’s curvature during the recovery and confirms the qualitative explanation of the operant phenomena of the preceding section. The model predicts a recovery that is about 1–2 seconds faster; this might be because the strip recovers in water, which may provide some resistance to the recovery motion.

The theoretical model provides an effective tool to help design the printed active composites. For example, the programming strain influences the residual strain stored in the programmed PACs. Using the same dimensions of the composite shown in Fig. 2a, we vary the prescribed strain and measure the curvatures of the sample in the recovery procedure. The length of the sample L=70mm and the programming temperature T_H_=70 °C, T_L_=0 °C. Figure 4b shows the initial curvature and the maximum curvature of the PAC as a function of the pre-stretch strain, using values from both experiments and theoretical predictions. It is clear that the curvature increases with the pre-stretch because the residual mismatch strain, which is the driving force of the bending, increases with the programming strain.

The effects of the volume fraction of the fibers are investigated by experiments and simulations. We vary the number of fibers in fiber family 1 (which has the higher T_g_ (in purple color)) to vary the volume fraction from 14.4% to 7.22%, 3.61% and 2.4%. Comparison between the experimental results and theoretical predictions of the initial and maximum curvatures are plotted as a function of the volume fraction of the fiber with higher T_g_ in Fig. 4c, which shows that when the volume fraction increases the maximum curvature increases monotonically; however, the initial curvature is nearly unchanged. We noticed that there is a deviation in the trend for the maximum curvature at lower volume fraction. This may be caused by the experimental errors such as the temperature accuracy, pre-strain error and measurement errors. We plot the error bars in this figure. Considering the experimental errors, we think the deviation is in a reasonable range.

### Printing self-folding and self-opening structures

As described above, by tuning the volume fraction of fibers and the prescribed strain, the bending of the SMP composites can be controlled. Here we show three examples of self-folding and opening structures that exploit this phenomenon and our understanding of it. All the designed structures are deformed in hot water at temperature of 70 °C and then cooled to ~0 °C.

**Figure 5:**
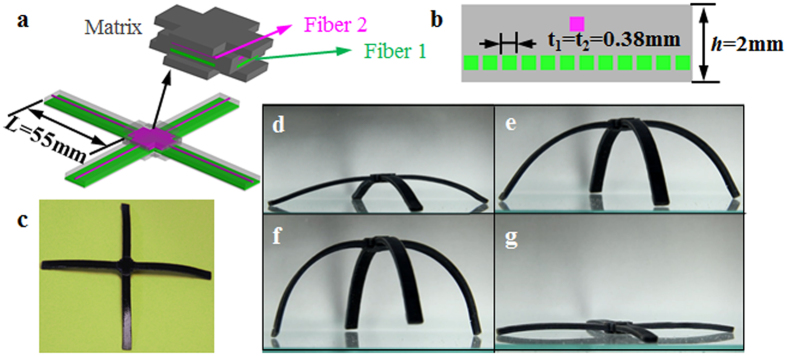
Self-assembling and disassembling trestle. (**a**) The design of the trestle. (**b**) The cross section of the composites strip. (**c**) The shape of the structure after programming. (**d**–**g**) The deformation behavior of the structure in the recovery process.

We first design a self-assembling and disassembling trestle. Figure 5a shows the design of the trestle, which has 4 identical active composite strips connected at the center. Figure 5b shows the cross-section of the composite strip. Every strip has one fiber with higher T_g_ (fiber 1) and twelve fibers with lower T_g_ (fiber 2) and the dimensions of the strip are 55 mm (length) by 6 mm (width) by 2 mm (thickness). The volume fractions of fiber 1 and fiber 2 are 1.2% and 14.4% respectively. The structure is stretched with a strain of 8% at 70 °C then cooled to 0 °C. After releasing at the low temperature, the sample bends slightly with a small curvature, as shown in Fig. 5c. When heated, the strips fold and the trestle stands up from the flat shape as shown in Fig. 5d–f. If we continue heating the sample, the trestle goes back to its flat shape (Fig. 5g). This design can be used as an active supporting trestle controlled by external heating.

**Figure 6:**
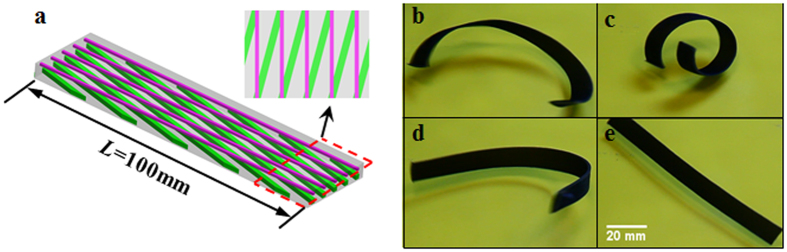
Active helix shape. (**a**) The details of the design. (**b**–**e**) The deformation behavior of the active helix in the recovery process.

We design and fabricate an active helix as shown in Fig. 6a. Here, we change the orientation of the fibers in the bottom layer. In the top layer the fiber (fiber 1) is 0.16 mm by 0.16 mm and the orientation is along the longitudinal direction of the PAC strip, while in the bottom layer the cross-section of the fiber (fiber 2) is 0.3 mm by 0.3 mm, and the fiber has an angle of 15° with respect to the longitudinal direction. The thickness of the sample is 1 mm. The corresponding volume fractions of the fibers in top and bottom layer are 2.6% and 9%, respectively. After the programming step, the printed flat strip bends as shown in Fig. 6b. When we increase the temperature, the sample bends and curls significantly and forms a helix shape and finally recovers to the flat permanent shape (Fig. 6b–e) due to the bend-twist coupling of the asymmetric composite laminate.

**Figure 7:**
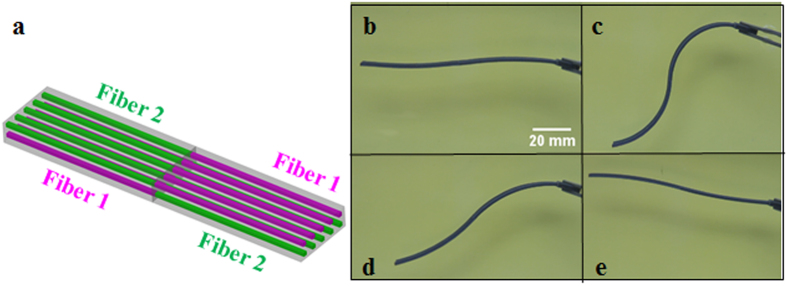
Active “wave” shape. (**a**) The design of the structure. The positions of fibers are exchanged in two segments. The figure is used for describing the design method, not to scale. (**b–e**) The deformation behavior of the active “wave” shape when heated to a temperature of 70 °C.

By alternating the position of fibers in the top and bottom layer, which is difficult to do with traditional composite laminates but straightforward with 3D printing, we can also create a “wave” shaped structure that can bend toward opposite directions. Figure 7a shows the design of the structure, and the dimensions of the cross-section of the structure are the same as Fig. 2a. The volume fraction of the fibers in both layers is 14.4%. The length of the sample is 100 mm and the programing strain is 10%. To create such actuation behavior, the sample is designed with two segments connected in the middle. The fibers’ orientation is parallel to the length direction. Figure 7b–e show results of the shape change of the PAC strip. We envision that if we arrange an array of these segments by changing the position and materials of the fibers, many ‘wave’ shapes can be achieved.

## Discussion

**Figure 8:**
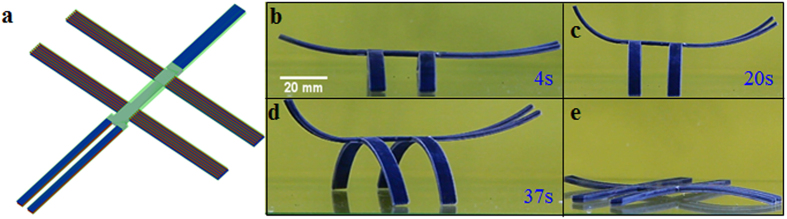
Mimicking an insect. (**a**) The design of the structure. The figure is used for describing the design method. Not to scale. (**b**,**c**) The deformation behavior of the active insect when put in the hot water with temperature of 30 °C. (**d**) Rotated view of the structure in hot water. (**e**) Recovered shape in 60 °C water.

**Figure 9:**
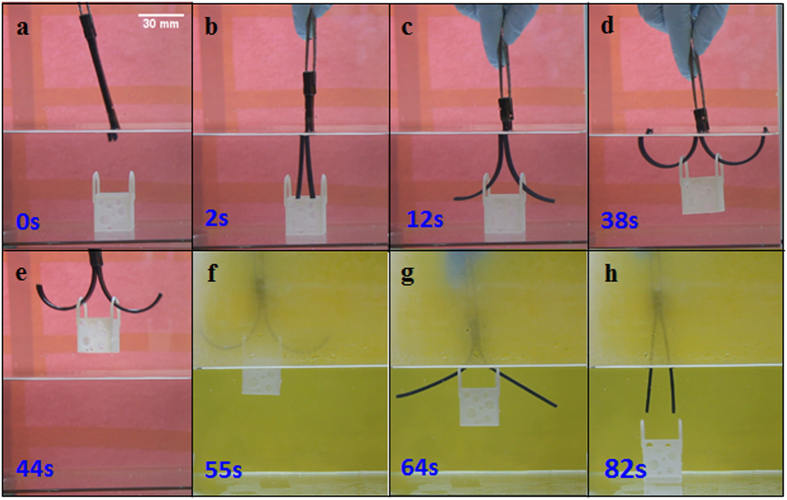
Smart hook. (**a**)The programmed hook. (**b**,**c**)The bending deformation of the structure under hot water with temperature of 30 °C. (**d**,**e**) A small box is lifted up from water. (**f-h**)Releasing the small box into another container. The water in the container is in 70 °C.

Using 3D printing technology to print shape memory polymers provides an easy and efficient way to manufacture active structures, and it can be used to create complex shapes tailored for specific applications. Here we further demonstrate two examples. The first one attempts to mimic an insect. As shown in Fig. 8a, the insect has a pair of antennae and a short tail. After simple biaxial tensile programming step, we heat the sample in 30 °C hot water (Fig. 8b,c). Under these conditions, the insect can stand up, and its tail and antennas turn up after 37s (Fig. 8c,d). If we further increase the temperature to 60 °C or higher, the insect will lie down on the ground (Fig. 8e). The second example is a smart hook (Fig. 9). In this design, two 3D printed composite strips are connected at the ends. After being stretched with 10% strain, cooled to 0 °C and relaxed under 0 °C water for 1 min, one end handle of the structure is cut off. Then the nearly flat sample can be used as a hook to lift a small box. We first put the sample into hot water with the temperature of 30 °C and the straight strips bend to form two half circular shapes (Fig 9b,c). Using the two half circular strips, we can lift a small basket from water (Fig. 9d,e). To release the box into another position, we increase the temperature of the sample to be higher than the T_g_s of the fibers (Fig. 9f–h). From these simple designs, we can see the great potential of using the 3D printed active materials in creating smart structures that can deform on demand by controlling the environment temperature.

3D printing is applied to design and create multi-shape memory composites. After being programmed by a simple one-step loading training process, the PAC is able to go through multiple shapes, depending on the temperature condition, and finally recover to the permanent shape. To understand the multi-shape memory behavior of the PACs and help the design of active structures with PACs, a theoretical thermo-mechanical model is established to investigate the curvature evolution during the shape recovery process. The influence of the volume fraction of SMP fibers and programming strain on the initial and maximum curvature of the PACs are studied by experiments and theoretical analysis. Using the design methods and theoretical predictions, we design and print several self-folding and opening structures by 4D printing to demonstrate the tremendous design freedom that is offered.

In this paper, we demonstrate the design strategy to take advantage of 3D printing to create novel shape changes, or the so-called 4D printing, which are pursed actively in the years. The design principles in this paper can be used to create active composite structures with more shape changes. However, one hurdle in implementing the designs with more shape changes is the limited choice of printed materials, which, in our cases, the few materials in Objet printer material library. However, we believe, with the rapid development of 3D printing, more printable polymers will become available; the design concept and methodology can be applied with new materials to fit the needs of specific applications. Although this paper is not for any specific applications, we can envision a few possible ones. For example, in biomedical application, the 30 °C is close to human body temperature, thus can be used to change into one shape and fulfill certain function; the shape can be further changed after a function is finished by taking out human body then heating up. For another example, we can envision our method can be applied to shape changing soft robots.

## Methods

### Fabrication of materials

All the samples are fabricated using an Objet 3D printer (Objet 260), which can mix two base materials to make digital materials with different mechanical properties. One of the two base materials is Tangoblack+, which is a rubbery material at room temperature, polymerized by urethaneacrylate oligomer, Exo-1,7,7-trimethylbicyclo hept-2-yl acrylate, methacrylate oligomer, polyurethane resin, and photoinitiator; the other one is Verowhite, which is a rigid plastic at room temperature, polymerized by isobornyl acrylate, acrylic monomer, urethane acrylate, epoxy acrylate, acrylic monomer, acrylic oligomer, and photoinitiator. During printing, the printer can mix the two materials with different component ratios to achieve the material with the user-desired mechanical properties. In the current printing system, users can only use a limited number of digital materials, as predefined by the printer’s processing program. The three digital materials used in this paper are TangoBlack plus (the matix), DM8530 (fiber 1) and DM9895 (fiber 2).

### Dynamic mechanical analysis test

Dynamic mechanical analysis (DMA) tests are performed on a DMA machine (model Q800, TA Instruments Inc, New Castle, DE, USA) in film tension mode. The material samples (dimension 10 mm × 3 mm × 1 mm) are first heated to 90 °C (60 °C for matrix material) on the DMA machine and stabilized for 10 minutes to reach thermal equilibrium. Then temperature is decreased from 90 °C ((60 °C for matrix material) to −30 °C (−50 °C for matrix material) with a rate of 2 °C/min. A preload of 0.001N is applied to keep the sample straight during the test. During the DMA tests, the strain is oscillated at a frequency of 1 Hz with peak amplitude of 0.1%. The experimental data during the cooling step are used to model the thermomechanical properties of the matrix and fibers.

**Figure 10:**
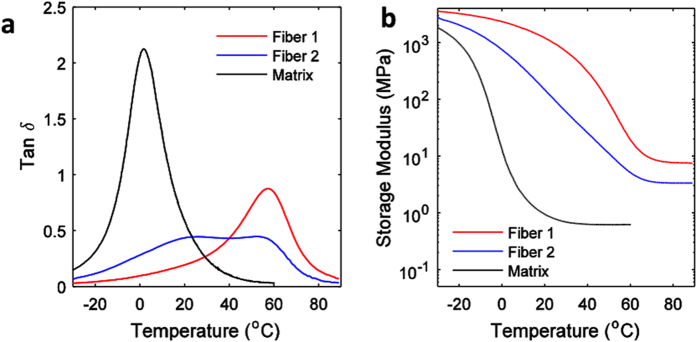
Dynamic mechanical analysis (DMA) test results of the SMP materials. (**a**) Tan δ and (**b**) storage modulus for three printed materials.

The DMA test results are used to characterize the viscoelastic properties of the printed materials. Figure 10 shows the DMA test results of the three SMP materials used in this paper. The glass transition temperature T_g_ is measured as the peak of the tan δ curve. For fiber 1, the T_g_ is ~57 °C; for matrix, the T_g_ is ~2 °C. Fiber 2 has a flat region in tan δ; we take the mid-point as the T_g_ which is ~38 °C.

### Theoretical model for the deformation behavior of the PAC strip

The bending behavior is induced by the strain mismatch between two layers due to the difference of the viscoelastic properties of the SMP materials. In our model, the composite contains two layers and both layers contain fibers and matrix. The whole set of thermomechanical loading steps shown in Fig. 1 are considered.

During the stretching step at high temperature T_H,_ the strain of the matrix and fibers is:


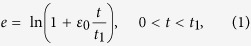


where *ε*_*0*_ is the prescribed strain during this step, *t* is the temporary time and *t*_1_ is the end time of this loading step.

In the cooling step, the temperature decreases from T_H_ to T_L_ during time period from *t*_*1*_ to *t*_*2*_. The thermal deformation during this procedure is:


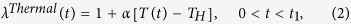


where *α* is the thermal expansion coefficient and *λ*^*Thermal*^ is the deformation due to the thermal expansion. Considering that the sample is constrained in this step, the total strain is kept unchanged. Therefore the mechanical deformation can be calculated as:


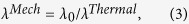


where *λ*_0_ is the stretch in the stretching step. Then the strain can be obtained as


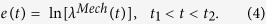


In the third step, the strain loading is released at low temperature T_L_ and the sample bends with a curvature *κ*(*t*). The Cartesian coordinates are set at the middle plane of the sample, the y-axis is in the thickness direction of the laminate, and the x-axis is in the width direction. After bending, the middle plane deforms Δ*λ*(0, *t*) = 1 + *ε*_*b*_(*t*) while the plane perpendicular to the y axis deforms Δ*λ*(*y*, *t*) = 1 + *ε*_*b*_(*t*) + *κ*(*t*)*y*. During bending, the total mechanical stretch is:


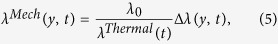


and the Hencky strain can be expressed as


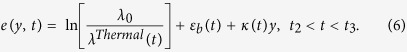


In the final step, the sample will be heated up to the prescribed temperature T_H_. Similar to the heating step, the thermal deformation is *λ*^*Thermal*^(*y*, *t*) = 1 + *α*[*T*(*y*, *t*) − *T*_*H*_], and the mechanical stretch and Hencky strain are


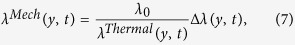


and


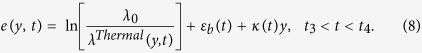


Note that in this step, as the time period of the heating process is quite short, the temperature field *T* is a function of not only time *t*, but also position *y*. It is reasonable to assume that the heat flux only flows along the thickness direction, since the thickness of the sample is much smaller than the width and length. One-dimensional heat transfer analysis is performed to calculate the temperature field *T*(*y*, *t*). By solving a one-dimensional boundary value problem:


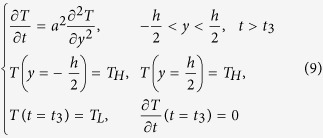


the temperature field is given by


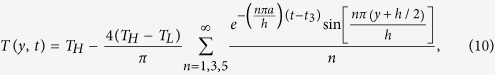


where 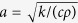, *k* is the coefficient of thermal conductivity, *c* is the specific heat capacity and *ρ* is the density.

To describe the bending deformation behavior of the SMP composites strip, the simple one dimensional Euler-Bernoulli beam theory is used. The simplicity is based on the simple bending deformation of our sample.

After releasing the constraint, the total external force and total external moment applied to the hinge are zero:


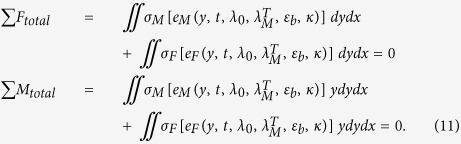


In Eq. (11), the stresses on the matrix *σ*_*M*_ and fibers *σ*_*F*_ can be calculated through the constitutive equation Eq. (S1) by incorporating the corresponding Hencky strain in Eqs (6 and 8). The variables *λ*_0_, *λ*^*Thermal*^ and *t* in Eqs (6 and 8) are calculable or measurable, but *ε*_*b*_ and *κ* are unknown variables to be calculated by solving Eq. (11).

## Additional Information

**How to cite this article**: Wu, J. *et al.* Multi-shape active composites by 3D printing of digital shape memory polymers. *Sci. Rep.* **6**, 24224; doi: 10.1038/srep24224 (2016).

## Supplementary Material

Supplementary Information
