# Supplementary material for: Multi-shape active composites by 3D printing of digital shape memory polymers
Source: Sci Rep. 2016 Apr 13;6:24224. doi: 10.1038/srep24224 (PMC4829848; doi:10.1038/srep24224)
Supplement: Supplementary Information [file srep24224-s1.pdf]

Supporting Information

Multi-shape active composites by 3D printing of digital shape memory polymers

Jiangtao Wu[1], Chao Yuan[2], Zhen Ding [3], Michael Isakov[1], Yiqi Mao[1], Tiejun Wang[2], Martin L. Dunn[3*], H. Jerry Qi[1]*

[1]*The George Woodruff School of Mechanical Engineering, Georgia Institute of Technology, Atlanta, GA 30332, USA*

[2]*State Key Laboratory for Strength and Virbration of Mechanical Structures, Department of Engineering Mechanics, Xi'an Jiaotong University, Xi'an 710049,China*

[3]*SUTD Digital Manufacturing and Design (DManD) Centre, Singapore University of Technology and Design, Singapore*

Table SI. The modeling parameters for three materials.

	Fiber 1		Fiber 2		Matrix	
**Branch**	E_{non} (Pa)	τ_i	E_{non} (Pa)	τ_i	E_{non}(Pa)	τ_i
E_1	1.7E+08	1E-07	3E+08	0.0001	1.96E+08	8.04E-06
E_2	1.88E+08	9.93E-07	2.75E+08	0.000657	2.02E+08	9.28E-05
E_3	2.12E+08	0.00001	2.96E+08	0.003872	2.4E+08	0.000827
E_4	2.39E+08	9.08E-05	3.05E+08	0.02	2.7E+08	0.006032
E_5	2.68E+08	0.00074	3.5E+08	0.1	2.92E+08	0.036577
E_6	2.93E+08	0.005374	3.78E+08	0.576863	3.32E+08	0.860502
E_7	3.08E+08	0.035368	2.92E+08	3.401616	3.03E+08	0.187428
E_8	2.91E+08	0.2	2.15E+08	20	1.41E+08	4.728029
E_9	2.85E+08	0.954957	1.47E+08	96.82391	13594410	83.13049
E_{10}	1.38E+08	3.182197	95213467	362.9461	505667.4	10000
E_{11}	1.62E+08	7.497457	63127650	1000	1158992	1848.755
E_{12}	1.78E+08	25.11365	62092100	2671.527	3453594	375.1723
E_{13}	1.53E+08	87.11596	52099306	7912.87	45511.49	935839.6
E_{14}	1.33E+08	283.7953	42374719	23498.79	50809648	20
E_{15}	1.22E+08	905.6253	35205449	71461.38	204851.1	67650.29
E_{16}	1.12E+08	3025.975	27897552	228551.6		
E_{17}	98095537	10000	20760769	726401		
E_{18}	83260945	32677.22	15532429	2277776		
E_{19}	65704556	96510.16	11281878	7091525		
E_{20}	59120212	267333.4	8305791	21997171		
E_{21}	51922181	773277.7	5959708	68236585		
E_{22}	44769327	2339554	4351312	2.08E+08		
E_{23}	34599493	7613180	3329757	6.41E+08		
E_{24}	21727122	26070126	2644468	2.07E+09		
E_{25}	9995279	1E+08	2196711	7.07E+09		
E_{26}	2916758	5.22E+08	1578065	2.4E+10		
E_{27}	957137.7	5.77E+09	107012.2	1E+11		
T_g (℃)	57		38		2	
T_{ref}(℃)	17		-3		-11	
C1	17.44		17.44		11.44	
C2	50.5		42.1		50.3	
AF_c/k	-24000		-23000		-20000	
E_{eq} （Pa）	7.50E+06		3.30E+06		0.6*10^6	

Constitutive model and parameter characterization

To describe the thermomechanical behavior of the SMP material, the multi-branch model is used, in which one equilibrium branch and several thermoviscoelastic nonequilibrium branches are arranged in parallel. Maxwell elements are used in the nonequilibrium branches to represent the relaxation behavior of the material, and the total stress of the material can be expressed as:

$$\sigma_{total} = E_{eq} e + \sum_{m=1}^{n} E_{non}^{m} \int_0^t \frac{\partial e}{\partial s} \exp\left[-\int_s^t \frac{dt'}{\tau_m(T)} \right] ds , \quad \text{(S1)}$$

where E_{eq} is the Young's modulus of the equilibrium branch, E_{non}^{m} and τ_m are the Young's modulus and temperature dependent relaxation time of the *m*-th nonequilibrium branch. According to the time temperature superposition principle (TTSP), τ_m can be calculated using the relaxation time τ_m^R at reference temperature:

$$\tau_m(T) = a^{Shift}(T)\tau_m^R , \quad \text{(S2)}$$

where $a^{Shift}(T)$ is the temperature dependent shifting factor. According to O'Connell and McKenna, the shifting factors can be calculated by combining the Williams–Landel–Ferry (WLF) equation[33] and Arrhenius-type equation [34]. When the temperature is higher than the reference temperature, the shifting factor can be expressed using the WLF equation:

$$\log\left[a^{Shift}(T)\right] = -\frac{C_1(T - T_{ref})}{C_2 + (T - T_{ref})}, T > T_{ref} , \quad \text{(S3)}$$

where C_1, C_2 and T_{ref} are the material parameters to be characterized. If the temperature is lower than the reference temperature T_{ref}, the shifting factor can be expressed by Arrhenius-type equation:

$$\log\left[a^{Shift}(T)\right] = -\frac{AF_c}{k^{Boltz}}\left(\frac{1}{T} - \frac{1}{T_{ref}}\right), T < T_{ref} , \quad \text{(S4)}$$

where A, F_c, k^{Boltz} are the material constant, configurational energy and Boltzmann's constant respectively.

The DMA test results in Fig. 2 are used to identify the above parameters, including E_{eq}, E_{non}^{m}, τ_m^R, C_1, C_2, and AF_c/k^{Boltz} AF_c/k . The storage modulus at 90 °C can be considered as the equilibrium modulus E_{eq} of each material, as the relaxation time at this temperature in each nonequilibrium branch is minimal. Employing the nonlinear regression (NLREG) method [35,36], E_{non}^{m}, τ_m^R, C_1, C_2, and AF_c/k^{Boltz}

AF_c/k . can be determined by fitting the $\tan\delta$ and storage modulus curves shown in Fig. 2. For the multi-branch linear model, the temperature dependent storage modulus $E_s(T)$, loss modulus $E_l(T)$ and loss factor $\tan\delta(T)$ can be respectively represented as

$$E_s(T) = E_{eq} + \sum_{m=1}^{n} \frac{E_m^{non}\omega^2\left[\tau_m(T)\right]^2}{1+\omega^2\left[\tau_m(T)\right]^2}, \tag{S5a}$$

$$E_l(T) = \sum_{m=1}^{n} \frac{E_m^{non}\omega\tau_m(T)}{1+\omega^2\left[\tau_m(T)\right]^2}, \tag{S5b}$$

$$\tan\delta(T) = \frac{E_l(T)}{E_s(T)}, \tag{S5c}$$

where ω is the test frequency.

The determined parameters are provided in the table in the supporting information. Figure S1 shows the comparison of the DMA curves between experiment and simulation for three testing materials. Good agreement indicates that these identified parameters can describe the thermomechanical behavior of the SMP materials very well.

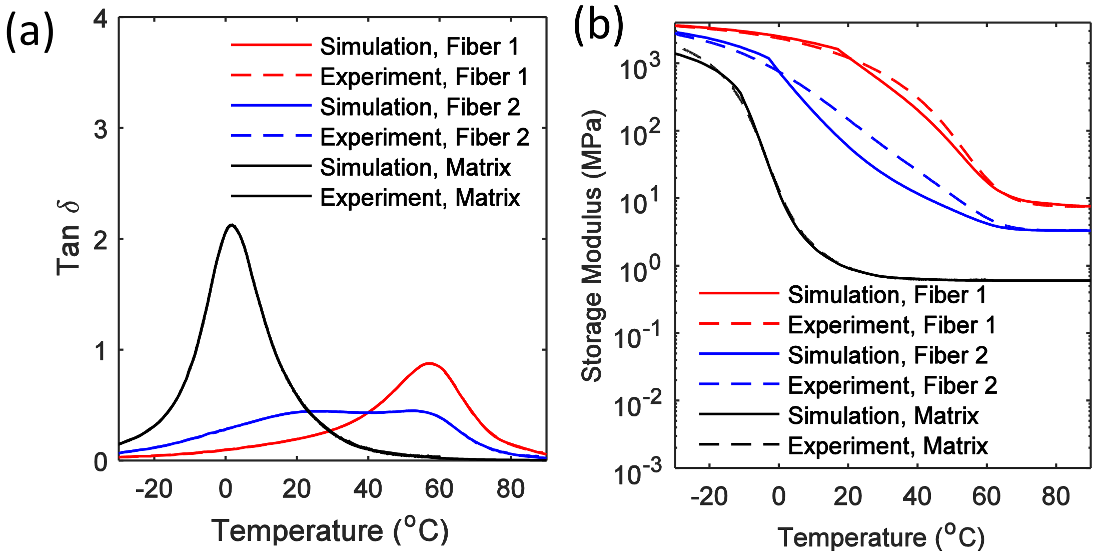

Figure S1. Comparison of the DMA curves between experiments and the simulation for three SMP materials.
